# New Medical School

**Published:** 1850-05

**Authors:** 


*New Medical School.*—A bill has been introduced into the Massachusetts Legislature to establish a new Medical School at Springfield, Mass.

